# Determinants of use of maternal health services in Nigeria - looking beyond individual and household factors

**DOI:** 10.1186/1471-2393-9-43

**Published:** 2009-09-15

**Authors:** Stella Babalola, Adesegun Fatusi

**Affiliations:** 1 Department of Health, Behavior and Society, Johns Hopkins University, Baltimore, USA; 2 Department of Community Health, College of Health Sciences, Obafemi Awolowo University, Ile-Ife, Nigeria

## Abstract

**Background:**

Utilization of maternal health services is associated with improved maternal and neonatal health outcomes. Considering global and national interests in the Millennium Development Goal and Nigeria's high level of maternal mortality, understanding the factors affecting maternal health use is crucial. Studies on the use of maternal care services have largely overlooked community and other contextual factors. This study examined the determinants of maternal services utilization in Nigeria, with a focus on individual, household, community and state-level factors.

**Methods:**

Data from the 2005 National HIV/AIDS and Reproductive Health Survey - an interviewer-administered nationally representative survey - were analyzed to identify individual, household and community factors that were significantly associated with utilization of maternal care services among 2148 women who had a baby during the five years preceding the survey. In view of the nested nature of the data, we used multilevel analytic methods and assessed state-level random effects.

**Results:**

Approximately three-fifths (60.3%) of the mothers used antenatal services at least once during their most recent pregnancy, while 43.5% had skilled attendants at delivery and 41.2% received postnatal care. There are commonalities and differences in the predictors of the three indicators of maternal health service utilization. Education is the only individual-level variable that is consistently a significant predictor of service utilization, while socio-economic level is a consistent significant predictor at the household level. At the community level, urban residence and community media saturation are consistently strong predictors. In contrast, some factors are significant in predicting one or more of the indicators of use but not for all. These inconsistent predictors include some individual level variables (the woman's age at the birth of the last child, ethnicity, the notion of ideal family size, and approval of family planning), a community-level variable (prevalence of the small family norm in the community), and a state-level variable (ratio of PHC to the population).

**Conclusion:**

Factors influencing maternal health services utilization operate at various levels - individual, household, community and state. Depending on the indicator of maternal health services, the relevant determinants vary. Effective interventions to promote maternal health service utilization should target the underlying individual, household, community and policy-level factors. The interventions should reflect the relative roles of the various underlying factors.

## Background

Approximately 536,000 maternal deaths occur annually, of which over 95% occur in sub-Saharan Africa and Asia [1]. Africa has the highest burden of maternal mortality in the world and sub-Saharan Africa is largely responsible for the dismal maternal death figure for that region, contributing approximately 98% of the maternal deaths for the region [1]. The lifetime risk of maternal death in sub-Saharan Africa is 1 in 22 mothers compared to 1 in 210 in Northern Africa, 1 in 62 for Oceania, 1 in 120 for Asia, and 1 in 290 for Latin America and the Caribbean [1]. Nigeria is a leading contributor to the maternal death figure in sub-Saharan Africa not only because of the hugeness of her population but also because of her high maternal mortality ratio. Nigeria's maternal mortality ratio of 1,100 is higher than the regional average [2]. With an estimated 59,000 maternal deaths, Nigeria which has approximately two percent of the world's population contributes almost 10% of the world's maternal deaths [3].

Scientific evidence has clearly established the inverse relationship between skilled attendants at birth and the occurrence of maternal deaths. Thus, the considerable variation in the maternal mortality estimates between different locations within the same region can be attributed, to a large degree, to the differences in the availability of and access to modern maternal health services [3]. The use of maternal health services also contributes to neonatal health outcomes as the health of the mother and the newborn is closely linked. Maternal complications in labor, for example, carry a high risk of neonatal death [4,5]. Three-quarters of neonatal deaths occur in the first week, and the highest risk of death is on the first day of life. Furthermore, the main direct causes of neonatal death, globally, are preterm birth (28%), severe infections (26%), and asphyxia (23%) [5]. This epidemiological picture underscores the contribution of the delivery process to neonatal deaths.

While available evidence indicates limited benefit from traditional antenatal care services, focused antenatal care provides opportunity for early detection of diseases and timely treatment. It also provides opportunities for preventive health care services such as immunization against neonatal tetanus, prophylactic treatment of malaria through the use of intermittent presumptive treatment approach, and HIV counseling and testing. Furthermore, antenatal care exposes pregnant women to counseling and education about their own health and the care of their children. Thus, antenatal care may be particularly advantageous in resource-poor developing countries, where health seeking behavior is inadequate, access to health services is otherwise limited, and most mothers are poor, illiterate or rural dwellers. With the strong positive association that has been shown to exist between level of care obtained during pregnancy and the use of safe delivery care, antenatal care also stands to contribute indirectly to maternal mortality reduction [6]. According to the 2003 Nigeria Demographic Health Survey (NDHS) [7], 37% of women who had births within the five years prior to the survey received no antenatal care for their most recent delivery while only 35.2% were assisted at delivery by a skilled attendant.

Several studies have assessed the individual and household determinants of utilization of maternal services. These studies have not yielded a consistent pattern of relationships between service utilization and individual and household predictors. In some cases, even when a strong association has been reported, such as in the case of the positive relationship between education and the use of skilled health attendants at birth, the extent and nature of the relationship are not uniform across social settings. For example, whereas studies in Peru [8] and Guatemala [9] showed that women with primary level education were more likely to utilize maternal health services compared to those without any formal education, some studies in Thailand [10] and Bangladesh [11] did not record any significant difference between the two educational groups. Distances to health services and rural locations have been generally reported to be strongly and negatively associated with the use of maternal health services [6]. Some studies conducted in Turkey [12] and southern India [13,14], however, did not show any significant difference in the use of antenatal care between urban and rural women. Association between age and service utilization has also been inconsistent across studies. Whereas many studies found a positive correlation between age and the use of skilled attendants at child birth [12,15-18], others have found a curvilinear relationship [19,20]. Religion has also shown variable pattern of association with service utilization, with significant association in some settings [21] but not in some others [13]. In contrast, parity has been consistently shown to be negatively correlated with the use of skilled attendants [10,14,15,19,22]. A number of studies have reported positive association between economic status and use of medical settings for delivery [13,10,16] whereas others have not found such an association [23,24].

One important inference from the review of existing literature is that the role of individual and household factors differs from one geographic and social setting to another. Thus, as several authors have aptly noted, the determinants of maternal health care service utilization vary across and within cultures [13,25].

It is reasonable to assume that utilization of maternal health services depends on individual and household factors, as well as factors operating at the community or policy levels. The review of extant literature however shows that very few studies have gone beyond individual and household factors to consider factors at the community and higher levels. The implication of this omission is that some determinants are inadvertently missed, leaving a serious research and programmatic lacuna. Secondly, failure to consider the role of factors operating beyond the household level in service utilization may result in serious bias in the estimates. Individuals are nested within families, which are in turn nested within communities. Methodologically, it is important to take this nested structure into account. This demands the use of multilevel modeling, which would calculate the standard errors more accurately and reduce the chance of misestimating the significance of variables, as some of the assumptions inherent in traditional regression methods are not valid for nested data [26].

Very few population-based studies have been carried out in Nigeria regarding determinants of maternal service utilization; most maternal health studies in the country have been institution-based. Most of the population-based studies were small-scale research, focusing on a handful of communities, usually small-sized rural communities [27-29]. Their geographic scope limits the applicability of their result on a large scale, particularly considering the complex multi-ethnic setting of Nigeria. In addition, most did not control for important confounding variables. Drawing from a nationally representative survey, this paper seeks to address the identified research gaps by examining the effect of individual, household, community and state-level factors on maternal care services utilization and employing strong analytical procedures. Specifically, we investigate the patterns and determinants of the utilization of the three dimensions of pregnancy-related care - ante-natal, delivery, and post-natal services.

## Methods

### Data

The data that we analyze in this paper derive from the 2005 National HIV/AIDS and Reproductive Health Survey (NARHS), a household survey designed to provide quantitative data for monitoring the impact of reproductive health interventions in Nigeria. The survey covered all the 36 states of Nigeria and participants were selected through a multi-stage probability sampling method. Details about the sampling have been provided elsewhere [30]. The original sample included 4,685 women (aged 15-49 years) and 5,396 men (aged 15-64 years); however, the sample included in the analyses reported in this paper was limited to the 2148 women who had a baby during the five years preceding the survey.

In addition to using data from the 2005 NARHS, we also accessed some state-level data published by National Bureau of Statistics for the year 2005 [31]. Specifically, we included information about the average number of residents to a Primary Health Care (PHC) facility in our estimated models. We use this variable as a proxy for the availability of maternal health services in the state.

### Measurement

We analyze the predictors of three indicators of use of maternal health services: use of antenatal care, delivery assisted by a trained medical personnel (doctor or nurse/nurse-midwife), and use of postnatal care services. We assess the predictors of each of these indicators separately and with reference to the most recent birth.

We examined the predictive value of a number of individual and household variables, including rank of the most recent birth, education, ethnicity, age at last birth, attitudes towards family planning, ideal family size and socio-economic status. We examined the role of three community level variables: type of place of residence (urban versus rural), media saturation in the local government area (LGA) of residence, and prevalence of the small family norm in the LGA of residence. At the state level, we assessed the role of the ratio of Primary Health Care (PHC) facilities to the population. In addition, we assessed random effects at the state level. We selected these predictors based on information from extant literature and because they were significant predictors in initial bivariate analyses of the data. We describe the various predictors in Table 1.

**Table 1:** Measurement of various predictors included in the estimated models

**Predictor**	**Measurement**
Rank of most recent birth:	We distinguish between mothers whose most recent birth is rank 1 or 2 (34.7%) and those whose most recent birth is of a higher rank.
Education:	Highest level of education attained is divided into four categories: none, primary, secondary, and post-secondary education.
Ethnicity:	We specifically recognized the largest ethnic groups in Nigeria (Hausa, Igbo, Yoruba, Fulani and Kanuri) while all the other ethnic groups are classified together.
Age at last birth:	The questionnaire did not include a direct question on the age at last birth; we computed this indicator by subtracting the child's age from the woman's current age and rounding the result to the nearest whole number.
Attitudes towards family planning:	We measure this indicator through reported approval of family planning.
Ideal family size:	We distinguish between the women who gave a numeric response to the question on ideal family size (52.0%) and those who gave non-numeric responses, such as "Up to God" (48.0%).
Household socio-economic status:	We constructed a scale for household socio-economic status from information on possession of specific household items and utilities, including refrigerator, radio, television, car, video player, cell phone, standby generator, electricity, fan, kerosene stove, pipe-borne water and water closet (Cronbach's alpha for internal reliability: 0.88). The resulting scale was divided into five quintiles.
Urban residence:	This variable was derived from the question on the type of place of residence; we compare urban residents with their rural counterparts.
Media saturation in the LGA of residence:	We operationalize this community-level variable through the mean level of exposure to the radio and the television for the people in the LGA of residence other than the index individual (the non-self mean). We divide the measure into three categories, viz.: low, medium and high levels of community media saturation based on the percentiles.
Prevalence of small family norm in the LGA of residence:	We measured this variable using the non-self mean of expressed preference for a small family (four children or less).
State of residence:	The NARHS 2005 survey took place in the 36 states and the Federal Capital territory. The state of residence was included as a random variable in the estimated models to represent unmeasured factors related to the socio-political and cultural context.
Number of people per PHC in the state of residence:	This information came from the statistics published by the National Bureau of Statistics for the year 2005.

### Analysis

Individuals are nested within households, households are nested within LGAs and LGAs are nested within states. In order to assess the roles of measured individual, household, community and state factors as well as unmeasured factors at the state level, we use multilevel modeling in this paper. The nature of nested data makes the use of traditional regression methods inappropriate: some of the assumptions inherent in traditional regression methods, including the assumption of independence among individuals within the same group and the assumption of equal variance across groups are not valid in the case of nested data [26].

We estimated a multilevel model that assessed the predictive values of measured individual, household, community and state factors (fixed effects) in addition to state-level random effects using the *gllamm *command in Stata [32]. For each of our three dependent variables, we estimated two models: an empty model that contains no covariates, and a full model that included fixed effects at the individual, household, community and state levels, and state-level random effects. The empty model allows us to verify if the magnitude of random effects at the state level justifies assessing random effects at that level. For all the estimated models, we evaluated the significance of the random effects using one-sided p-values rather than simple Wald tests since the null value is on the border of the parameter space [33,34].

## Results

About three-fifths (60.3%) of the respondents used antenatal services at least once during their most recent pregnancy. The percentage of last births whose delivery was assisted by qualified medical personnel (doctor, nurse or nurse-midwife) was 43.4% while only two fifths (41.2%) received postnatal care (Table 2).

**Table 2:** Variations in indicators of use of maternal and child health services, by selected individual, household and community characteristics.

**Characteristics**	**n**	**Percent reporting use of:**		
		
		**Antenatal care**	**Medical personnel at delivery**	**Postnatal care**
*Individual Factors*				
Education				
None	1013	37.1	17.7	19.3
Primary	537	74.3	51.5	49.3
Secondary	501	84.6	75.8	67.6
Post-secondary	107	95.3	93.4	83.2
Age at Last Birth				
15 -- 19	351	46.7	29.0	28.8
20 -- 24	546	58.2	37.5	36.1
25 -- 29	546	69.2	53.5	50.3
30 -- 34	367	68.1	50.9	49.0
35 -- 39	218	56.4	43.6	40.8
40 +	130	52.3	43.1	36.1
Ethnic Group				
Hausa	656	37.5	15.2	18.0
Yoruba	313	91.4	81.4	70.9
Igbo	221	87.3	78.7	73.7
Fulani	136	41.9	17.6	27.2
Kanuri	66	27.3	22.7	24.2
Others	766	65.4	48.2	43.5
Child's rank of birth				
1 -- 2	750	64.8	49.3	45.7
3 +	1408	57.9	40.2	38.8
Attitudes towards family planning				
Approve	1097	77.7	61.4	57.9
Disapprove	1061	42.2	24.7	23.9
Ideal family size				
Provided a numeric response	1122	74	61.0	55.1
Provided a non-numeric response (Up to God, etc.)				
	1036	45.4	24.3	26.0
*Household Factors*				
Household socio-economic status				
Very poor	440	27.9	13.2	17.0
Poor	466	44.0	24.9	22.3
Medium	394	62.4	39.8	38.8
Rich	431	80.0	64.7	58.4
Very rich	427	89.4	76.6	71.4
*Community Factors*				
Type of Place of Residence				
Rural	1495	49.3	32.1	31.4
Urban	663	84.9	68.8	63.3
Community media saturation				
Low	858	35.1	18.4	18.4
Medium	692	67.6	45.2	45.8
High	608	87.5	76.6	68.1
Prevalence of small family norm in community				
Low (0 -- 10%)	927	35.7	15.6	19.6
Medium (11 -- 30%)	663	68.5	48.7	45.1
High (< 30%)	568	90.8	82.6	71.8
*State-level Factors*				
Average number of people to a PHC in the state of residence				
Small (< 5500)	737	71.5	52.4	47.5
Medium (5500 -- 9000)	705	60.0	46.8	44.2
Large (> 9000)	716	49.0	30.8	31.7
All Respondents	2158	60.3	43.4	41.2

Source: Nigerian National HIV/AIDS and Reproductive Health Survey, 2005; Data on the ration of people to a Primary Health Center (PHC) came from the 2006 Core Welfare Indicator Questionnaire Survey, a national survey conducted by the Nigeria Federal Bureau of Statistics.

### Bivariate analysis

Table 2 shows variations in the three indicators of maternal health service utilization by selected socio-demographic, household, community and state factors. The results show that for each of the three indicators, there are significant differences by education, age at last birth, ethnicity, child's rank of birth, attitudes towards family planning, and ideal family size. There are also significant variations in the indicators by household socio-economic status, urban residence, community media saturation, prevalence of the small family norm in the LGA of residence, and the ratio of PHC to the population in the state of residence. For example, the three indicators of use increase steadily with education and household socio-economic status. In contrast, the indicators of use decrease by the child's rank of birth and are lower for women who gave non-numeric fertility ideals than their peers who reported gave numeric family size ideals. The relationship with age at last birth does not appear to be linear as the data show that the women most likely to use the antenatal care, medical personnel for delivery or postnatal care are those aged 25 - 34 years. In addition, the use of these services is more common among the women who approve of family planning compared to their peers who did not; urban women are also more likely to report use of the services compared to rural women. Yoruba, Igbo and minority women reported a significantly higher use of the services than Hausa, Fulani or Kanuri women.

Use of these services also increases steadily with community media saturation and the prevalence of the small family norm in the LGA of residence. In contrast, the three indicators decrease as the average number of people per PHC in the state of residence increases.

### Multilevel models

Obviously, the bivariate relationships indicated by the data on Table 2 can be due to interrelationships among the various measured characteristics as well as to unmeasured characteristics at the state level. We therefore used multilevel modeling to determine the predictors of maternal health services utilization and parse the variance in use into its fixed and random components. In the multilevel model, state of residence is modeled to be random.

We started with an empty, intercept-only model to test the null hypothesis that state-level variance in maternal health services utilization is zero and to assess if our data justify the decision to assess random effects at the state level. The results presented in Table 3 show that for each indicator of maternal health services utilization there is considerable between-states heterogeneity. For example, for antenatal care, the state-level variance in the empty model is large and significant pointing to considerable differences in use across states. The conditional intra-class (ICC) correlation in the empty model for antenatal care indicates that 36.8% of the total variance in use of antenatal care is attributable to the differences across states; in other words, use of service cluster significantly by state. We find similar results for assisted delivery and use of postnatal care (Table 3)

**Table 3:** Parameter coefficients for the multilevel model for various indicators of use of maternal and child health services -- Empty model, no covariates.

	**Antenatal care**	**Medical personnel at delivery**	**Postnatal care**
***Random Effects***			
State Level Variance	1.92*** (0.50)	2.17*** (0.54)	1.67*** (0.44)
Rho -- Intra-class correlation	0.368	0.397	0.337
Log-likelihood	-1165.64	-1125.421	-1221.41
AIC	2335.3	2254.8	2446.8

Source: Nigerian National HIV/AIDS and Reproductive Health Survey, 2005

Notes: *** p < 0.001;

We now turn our attention to the results of the full models that assess the roles of predictors at the various levels (Table 4). Since there are some differences in the predictors of the specific indicators of use, we present the findings separately for each indicator.

**Table 4:** Results of the multilevel analysis of the predictors of indicators of use of maternal and child health services.

**Characteristics**	**n**	**Odds Ratio (Std. Error)^a^**		
		
		**Antenatal care**	**Medical personnel at delivery**	**Postnatal care**
***Fixed Effects^b^***				
*Individual Factors*				
Education				
None RC)	1013	1.00	1.00	1.00
Primary	537	1.88*** (0.30)	1.69*** (0.28)	1.65*** (0.25)
Secondary	501	2.01*** (0.43)	3.01*** (0.60)	2.06*** (0.38)
Post-secondary	107	5.03** (2.64)	10.68*** (4.88)	3.50*** (1.15)
Age at last birth in single years	2158	1.18** (0.07)	1.08 (0.07)	1.13* (0.07)
Square of age at last birth	2158	0.99** (0.001)	0.99 (0.001)	0.99* (0.001)
Ethnic Group				
Hausa RC)	656	1.00	1.00	1.00
Yoruba	313	1.22 (0.51)	1.62 (0.58)	1.57 (0.52)
Igbo	221	2.09§(0.87)	3.76**** (1.49)	2.10* (0.78)
Fulani	136	0.68 (0.18)	0.77 (0.25)	1.22 (0.33)
Kanuri	66	0.67 (0.34)	1.72 (0.90)	0.97 (0.50)
Others	766	1.35 (0.32)	2.04** (0.53)	1.55§(0.38)
Child's rank of birth				
3 + (RC)	750	1.00	1.00	1.00
1 -- 2	1408	1.17 (0.19)	1.22 (0.20)	1.10 (0.16)
Attitudes towards family planning				
Disapprove (RC)				
Approve	1097	1.00	1.00	1.00
	1061	1.64*** (0.22)	1.28§(0.17)	1.58*** (0.20)
Ideal family size				
Provided a non-numeric response (Up to God, etc.) (RC)				
Provided a numeric response	1036	1.00	1.00	1.00
	1122	1.14 (0.16)	1.71*** (0.24)	1.47** (0.19)
*Household Factors*				
Household socio-economic status				
Very poor (RC)				
Poor	440	1.00	1.00	1.00
Medium	466	1.53* (0.27)	1.88** (0.43)	1.01 (0.20)
Rich	394	2.48*** (0.48)	2.72*** (0.62)	1.69** (0.34)
Very rich	431	3.76*** (0.87)	4.27*** (1.07)	2.46*** (0.55)
	427	5.86*** (1.69)	4.34*** (1.23)	3.02*** (0.76)
*Community Factors*				
Type of Place of Residence				
Rural (RC)				
Urban	1495	1.00	1.00	1.00
	663	2.36*** (0.48)	1.69** (0.33)	1.63** (0.29)
Community media saturation				
Low (RC)				
Medium	858	1.00	1.00	1.00
High	692	1.51* (0.25)	1.44* (0.27)	1.51* (0.26)
	608	1.29 (0.36)	2.17** (0.58)	1.20 (0.30)
Prevalence of small family norm in community				
Low (< 11%) (RC)				
Medium (11 -- 30%)	927	1.00	1.00	1.00
High (< 30%)	663	1.39 (0.30)	1.50§(0.33)	1.14 (0.24)
	568	1.91* (0.58)	1.85* (0.53)	1.49 (0.40)
*State-level Factors*				
Average number of people to a PHC in the state of residence				
Small (< 5500) (RC)				
Medium (5500 -- 9000)				
Large (> 9000)	737	1.00	1.00	1.00
	735	0.63 (0.24)	0.71 (0.24)	0.87 (0.30)
	716	0.42* (0.16)	0.41** (0.14)	0.66 (0.23)
***Random Effects***				
State Level Variance		0.70** (0.23)	0.56** (0.19)	0.60** (0.20)
Residual intra-class correlation		0.183	0.152	0.160
Log-likelihood		-939.50	-881.86	-1052.80
AIC		1931.01	1815.73	2157.61

Source: Nigerian National HIV/AIDS and Reproductive Health Survey, 2005; Data on the ration of people to a Primary Health Center (PHC) came from the 2006 Core Welfare Indicator Questionnaire Survey, a national survey conducted by the Nigeria Federal Bureau of Statistics.

Notes: § p < 0.1; * p < 0.05; ** p < 0.01; *** p < 0.001

RC = reference category

Standard errors are in parenthesis

^a^Model with fixed effects at the individual, household, community and state levels, and random effects at the state level;

^b^fixed effects expressed as odds ratio

#### Antenatal Care

The most significant individual-level predictors of use of antenatal care services are education, age at the birth of last child, and attitudes towards family planning (Table 4). The odds of reporting use of antenatal care services increase steadily with education such that the women with post-secondary education are five times as likely to report service use as their counterparts with no formal education. Approval of family planning, a variable reflecting modern as opposed to conservative ways of thinking, is associated with a 64% increase in the odds of reporting use. The relationship between age at the birth of the last child and use of antenatal care services appears to be curvilinear. The negative coefficient associated with the square of age indicates that use of antenatal services initially increases with age up to a threshold and decreases thereafter.

Household socio-economic status is positively related with use of antenatal services such that the odds of reporting use are almost six times as high among women from the richest households compared to their counterparts from the poorest households. The three community level variables included in the model turn out to be significant predictors of antenatal services utilization. Living in an urban community increases the odds of antenatal service utilization more than twofold. The data show a rather curious relationship with community media saturation: compared to low media saturation, a high level of community media saturation does not seem to make a difference whereas medium level does. In contrast, concerning the small family norm, it appears that only a high level of prevalence makes a significant difference.

The data further show that the larger the number of residents served by a PHC in the state, the less the odds that women would use antenatal care services. Finally, state-level random effects are significant; the residual intraclass correlation is still appreciably large, indicating that even after controlling for individual, household and community factors, there is still considerable clustering of antenatal service utilization at the state level.

#### Skilled Assistance at Child Birth

The individual-level variables that predict use of skilled (medical) personnel for delivery include education, ethnicity, and family size ideals (Table 4). As we saw with the use of antenatal care, the use of medical personnel for delivery increases steadily with education. This indicator of service use is also a function of ethnicity: compared to women of Hausa descent, Igbo and minority women are significant more likely to report use of skilled assistance for delivery. Providing a numeric ideal family size is also associated with increased odds of using medical personnel at delivery.

Furthermore, the odds of reporting this indicator of use increase monotonically with household socio-economic status and are higher for urban residence compared to their rural peers. In addition, both community media saturation and the prevalence of the small family norm in the community present a graduated, dose-response relationship with use of medical personnel for delivery. State-level random effects are also significant and there is evidence of clustering at the state level even after controlling for individual, household and community variables.

#### Postnatal Care

At the individual level, education, age at the birth of the last child, ethnicity, approval of family planning and family size ideals are the strongest predictors of postnatal care (Table 4). Specifically, as we noticed with the two previous indicators, use of postnatal care increases consistently with education. The odds of using postnatal care are also significantly higher for women who approve of family planning and who report numeric family size ideals compared to their counterparts who did not report these attitudes. Although not as strong as what we observed for antenatal use, age presents a curvilinear relationship with use of postnatal care.

At the household level, socio-economic status is a significant positive predictor. Two community-level variables - urban residence and community media saturation - are significant predictors but the prevalence of the small family norm is not. Unlike what we observed for the two previous indicators of service utilization, the indicator of health services availability in the state does not appear to make a significant difference for use of postnatal care. Nonetheless, the state-level random effects are significant with unmeasured state-level factors accounting for 16% of total variance in the use of postnatal care.

## Discussion

This study is based on the NARHS, which involved a nationally representative population sample, and marks a departure from most of the previously reported studies on maternal health services utilization in Nigeria in terms of its national coverage. In addition, unlike most previous studies, we covered the three dimensions of pregnancy-related care - antenatal, delivery and postnatal services.

Our results show that the level of utilization of orthodox health care facilities for maternal care among women in Nigeria is low. Indeed utilization of maternal health care services is lower in Nigeria than in many countries in sub-Saharan Africa. For example, whereas we found that 60.3% of Nigerian mothers utilized antenatal care services during their last birth, the comparative figures were 88.0% for Benin (2006 DHS), 72.8% for Burkina Faso (2007 DHS), 83.4% for Cameroon (2004 DHS), and 91.9% for Ghana (2003 DHS) [35].

Similarly, the indicators of skilled assistance during delivery and use of postnatal care are considerably lower in Nigeria than in most African countries. A recent UNICEF report [36] shows that regarding skilled assisted delivery, only Burundi, Chad, Eritrea, Ethiopia, Niger and Somalia performed more poorly than Nigeria in sub-Saharan Africa.

The finding that utilization of antenatal services is higher than use of skilled assistance during delivery is consistent with the results of previous studies conducted in Nigeria [29,37] and elsewhere [38-40]. One of the reasons that have often been advanced for the lower coverage of skilled and institutional delivery compared to antenatal care coverage is the unpredictable nature of the onset of labor in the face of difficulty in accessing health facilities in resource-poor environments. Many rural communities in sub-Saharan Africa are examples of such environments, with the characteristic poor road networks, limited transportation means and underserved population in terms of health facilities. Our study would support such an explanation considering that the average number of residents per PHC is a more significant predictor of use of skilled assistance for delivery than of use of antenatal care.

The poor staffing of the health facilities, particularly the primary health care facilities, which makes it difficult to guarantee 24-hour availability of services had also been reported as a factor that discourages women in Nigeria, even when they had received antenatal care services, to seek medical services when labor commences [41]. The role of traditional and religious beliefs as well as the perception of women with regards to comparative efficacy of the medical versus traditional birth attendants may also be contributory to failure to have skilled attendants at birth. As Addai [42] pointed out, modern (medical) and indigenous maternal health care services coexist in most African communities, particularly in rural areas, and women may have to choose between the two options. Some previous studies had reported that many Nigerian women, particularly those in rural areas, rate the services of the traditional birth attendants (TBAs) as being of higher quality than that of medical healthcare practitioners, particularly with regards to interpersonal communications and relationships [41,43]. TBAs have been reported to be more considerate and to provide more compassionate care. Women in rural Guatemala have similarly been reported as being less likely to deliver in medical settings because of lack of social support provided by health-care professionals compared with traditional midwives [23]. Furthermore, Falkingham [24] reported that despite the fact that medical services were accessible and free of charge, women in Tajikistan prefer to deliver at home because they perceive available medical services to be of low quality and unsafe. Economic reason also ranks strongly in the preference of some Nigerian women for TBAs as their services have been reported to be more affordable. Additionally, TBAs may offer a more convenient user-charges system that allows payment to be spread over a period of time or even to be made in kind [44].

Our finding regarding the significant positive association between education and each of the three indicators of maternal services use agrees with previous reports [8,12,45,46]. Education serves as a proxy for information, cognitive skills, and values; education exerts effect on health-seeking behavior through a number of pathways [47]. These pathways include higher level of health awareness and greater knowledge of available health services among educated women, improved ability of educated women to afford the cost of medical health care, and their enhanced level of autonomy that results in improved ability and freedom to make health-related decisions, including choice of maternal services to use [10,12,48]. Educated mothers are more likely to take advantage of public health-care services than other women [49,50]. Education may also impart feelings of self-worth and confidence as well as reduce the power differential between service providers and clients, thereby reducing the reluctance to seek care [51,52].

The absence of a statistically significant association between the child's rank of birth and maternal services utilization among Nigerian women is surprising. Previous studies have found a strong negative association between parity and maternal services utilization [38,46,53,54].

Our finding with regards to the association between ethnicity and service utilization is an interesting one. Whereas ethnicity seems to make no significant difference for use of antenatal care, it does for use of skilled assistance and postnatal care. For these two indicators of use, the Fulanis, and the Kanuris (in the north) are not statistically different from the Hausas (in the north). In contrast, the level of service utilization was significantly higher among the Igbos (in the south) and the "minority" tribes compared to the Hausas. The pattern is consistent with the general picture of wide regional disparity in health status in Nigeria's diverse and multi-ethnic setting as has been reflected, for example, in the NDHS [7]. Perhaps more than other factors, this result reflects the influence of culture. An analysis of the social context of childbirth among the *Hausas *of Northern Nigeria, for example, has highlighted the strong influence of cultural beliefs and practices on childbirth and related fertility-related behaviors, and their significant contribution to the maternal morbidity and mortality picture [55]. In addition to the fact that a high proportion of teenage girls are married out to much older men, sometimes as early as 9 or 10 years of age, based on religious/cultural beliefs, cultural norms restrict women from readily seeking health-related assistance in pregnancy and childbirth. As Wall [55] noted, "*Kunya*, or 'shame' plays an extremely important role in *Hausa *childbirth, particularly in the first pregnancy. The newly pregnant girl should not draw attention to her gravid state, and all mention of the pregnancy should be avoided in conversation and action. This social pressure to remain 'modest' may well prevent her from asking questions about childbirth, and creates a major barrier to her seeking skilled assistance for delivering in hospital. As Wall further note, the pregnant girl's "mother, other relatives, and a local midwife usually stay with her during labor, but her *kunya *and her fear may be so great that she does not say anything until labor is well advanced." (p. 353). If there is nobody immediately available, it is unlikely that the girl in labor will send for someone, as "*kunya*" will prevent her from saying anything. Moreover in the cultural context of the Hausas, delivering her first child alone - unattended to by anyone - is viewed with pride.

Whereas some previous Nigerian studies had reported a significant relationship between age and maternal services utilization [56,57], others had shown no such difference [28,58]. We found no significant relationship between age and use of skilled assistance. For the other two indicators, we found a curvilinear relationship, such that women in the middle childbearing ages are more likely to used maternal services compared to their peers in the early or late childbearing ages. This finding agrees with the report of Obermeyer and Potter [19] and Gage [20].

Expectedly, fertility-related attitudes, which are reflected in our study by attitudes towards family planning and notion about the ideal family size, have significant relationship with maternal health services utilization. Favorable attitudes towards family planning and a clear notion about what constitutes an ideal smaller family size reflect less conservative behavior and more openness to modern health-related concepts and services.

At the household level, we found socio-economic status to be a significant predictor: for each of the three indicators, use of maternal services increases steadily with socio-economic status. Studies elsewhere have also documented positive relationship between economic status and early antenatal care use [16,58,59], delivery in medical settings [13,14,16], and utilization of postnatal services [60,61].

A major focus of this study is to go beyond individual and household factors and investigate the effects of community and state level factors on maternal care services utilization. At the community level, urban residence was consistently associated with increased odds of service utilization. This finding is in consonance with previous studies which have reported a significantly higher use of services in urban compared to rural areas in Nigeria [7,22,43,62] and elsewhere [13,16,63,64]. The other two community factors assessed - community media saturation and the prevalence of the small family norm in the community - are expectedly significant predictors of service utilization. Note however that the small family norm was not significant for postnatal care. The reason for this finding is not clear.

At the state level, we found that the ratio of PHC to the population was a significant predictor for use of antenatal care and skilled attendance at delivery, but not for postnatal care. The relationships are such that the larger the number of residents to a PHC the less the odds of using the services. This negative relationship is understandable since the more people a PHC serves, the more likely it is that access to the services would be difficult and the quality of services received poor.

Finally, we found that the random effects of the state of residence on each of the three indicators of maternal care service utilization are significant. Substantively, this finding shows that unmeasured factors operating at the state of residence level play a significant role in determining utilization of maternal health services beyond the influence of individual, household and community factors.

The findings from this study have implications for evidence-based programming. Collectively, the findings highlight the need for programs to adopt a multi-level approach and address the factors affecting maternal health services utilization at various levels - individual, household and community. More specifically, programs need to explore effective ways of increasing service utilization among lowly educated and poor women who are the least likely to use maternal health services. Evidence from elsewhere have shown that access to services and cost are serious barriers to service utilization among the poor [65,66]. As Fotso et al. surmised, it is not enough to increase the availability of services, making such services affordable to the poor is a necessity [67].

The strong role of community-level normative factors point to the need for interventions that target social norms. For example, using the media to disseminate consistent messages promoting the use of maternal health services could help to increase discussion of these issues within the community, a relevant step towards changing prevailing negative norms. Also relevant are efforts that involve community leaders and other key persons as agents of change. The findings that the prevalence of the small family norm in the community and personal fertility-related attitudes are associated with differences in service utilization suggest that promoting the use of family planning may ultimately help to foster the utilization of other maternal health services. In other words, programs that seek to promote the small family norm and change attitudes that are unfavorable towards family planning are relevant.

The significant state-level random effects that our study found demonstrate the need to contextualize efforts aimed at promoting maternal service utilization. There are obviously some unmeasured factors at the state level that predict service utilization. An effective strategy should be state-specific and seek to identify and address state-level factors that affect service utilization.

This study has some limitations that should be noted. First, the NARHS, the source of data for our study, was based on the self-report of respondents, and provided no validation of obtained information with any objective source such as health facility cards. The validity of self-reported behavior constitutes a concern in the literature, but it is logical to assume that biases are less likely in pregnancy-related events as compared to sensitive issues such as sexual behavior and drug abuse. Social desirability bias may also be an issue in cases that women feel they need to respond in a way expected of them. The comparability of our results with that of NDHS with regards to antenatal care use, for example, suggests that such bias is not likely to have affected our findings in any significant way.

Second, some known predictors of service utilization are obviously missing from our analyses. For example, availability of maternal health services within the immediate locality of respondents and the distance of respondents to such health services could have contributed to the picture of utilization pattern. Unfortunately these variables were not available in the NARHS. Although we included the state-level measure of PHC density (the number of residents to a PHC) in our analyses, the extent to which this variable is a good proxy for individual-level variable is uncertain.

Third, the study relied on cross-sectional data with the attendant potential selectivity and endogeneity bias. There is a possibility that the relationships that we found in our study are due to the influence of unmeasured individual and community-level variables that are associated with both the dependent and independent variables in our estimated models. It is also possible that the observed relationships reflect reverse causation or are due to measurement error. There are analytic methods to adjust for endogeneity bias in cross-sectional data (e.g., propensity score matching, bivariate probit regression, multivariate probit regression and instrumental variable regression) [68]. Nonetheless, adjusting for endogeneity is beyond the scope of this paper.

## Conclusion

Factors influencing maternal health services utilization operate at various levels - individual, household, community and state. While education, socio-economic level, and urban residence are consistently strong predictors of all the maternal health services considered in this study, other determinants of service utilization generally vary in magnitude and level of significance by the type of maternal service - ante-natal care, skilled attendant at birth, and postnatal care. To be optimally effective, interventions to promote maternal health service utilization need to take these findings into consideration: they should target the underlying individual, household, community and state-level factors that are relevant to each type of maternal health service. It is particularly important for interventions to explore effective ways of increasing service utilization among lowly educated and poor women in rural areas who are the least likely to use maternal health services.

## Competing interests

The authors declare that they have no competing interests.

## Authors' contributions

SB and AF were equally responsible for designing the study and drafting the manuscript. SB performed the statistical analyses while AF contributed the literature review.

## Pre-publication history

The pre-publication history for this paper can be accessed here:


